# Effect of Cow Dung Additions on Tropical and Mediterranean Earth Mortars-Mechanical Performance and Water Resistance

**DOI:** 10.3390/ma17122885

**Published:** 2024-06-13

**Authors:** Raphael N. Pachamama, Paulina Faria, Marco A. P. Rezende, António Santos Silva

**Affiliations:** 1 School of Architecture, PPG-ACPS, Federal University of Minas Gerais, Belo Horizonte 31270-901, MG, Brazilmarco.penido.rezende@hotmail.com (M.A.P.R.); 2 CERIS, Department of Civil Engineering, NOVA School of Science and Technology, NOVA University Lisbon, 2829-516 Caparica, Portugal; 3 National Laboratory for Civil Engineering, Av. Brasil, 101, 1700-066 Lisboa, Portugal; ssilva@lnec.pt

**Keywords:** adhesion, bio-stabilization, cattle manure, clay-based building, cracking, DIN 18947, earth plaster and render, performance under water, soil, strength

## Abstract

Cow dung (CD) is a material that has been used for millennia by humanity as a stabilizer in earth building techniques in vernacular architecture. However, this stabilization has been little addressed scientifically. In this study, the effect of CD additions was assessed on earth mortars produced with one type of earth from Brazil and two other types from Portugal (from Monsaraz and Caparica). The effect of two volumetric proportions of CD additions were assessed: 10% and 20% of earth + sand. The German standard DIN 18947 was used to perform the physical and mechanical tests, and classify the mortars. In comparison to the reference mortars without CD, the additions reduced linear shrinkage and cracking. An increase in flexural and compressive strengths was not observed only in mortars produced with earth from Monsaraz. In mortars produced with the earth from Caparica, the addition of 10% of CD increased flexural strength by 15% and compressive strength by 34%. For mortars produced with the earth from Brazil, the addition of 10% of CD increased these mechanical strengths by 40%. The increase in adhesive strength and water resistance promoted by the CD additions was observed in mortars produced with all three types of earth. Applied on ceramic brick, the proportion of 10% of CD increased the adherence by 100% for the three types of earth. Applied on adobe, the same proportion of CD also increased it more than 50%. For the water immersion test, the CD additions made possible for the mortar specimens not to disintegrate after a 30 min immersion, with the 20% proportion being more efficient. The effects of the CD on mechanical performance, including adhesion, were more significant on the tropical earth mortars but the effects on water resistance were more significant on the Mediterranean earthen mortars. CD has shown its positive effects and potential for both tropical and Mediterranean earthen plasters and renders tested, justifying being further studied as an eco-efficient bio-stabilizer.

## 1. Introduction

Earth is a striking material in Brazilian, Portuguese and many other countries’ vernacular architecture. Over the last decades, much research has been promoted worldwide not only to improve the conservation of that heritage but also to gain knowledge to support the use of earth in new buildings, mainly due to environmental concerns [1]. It is possible to find a wide variety of knowledge about earth construction technologies, methods, materials and stabilizing additions. In earth buildings, earth mortars are essential for many applications, namely for adobe production, masonry unit bedding, cob production, screeds, plastering and rendering, namely coating the surface of walls to protect them from mechanical impacts and rain action. For tropical countries with large populations and a lack of housing, like Brazil, research and improvements in earth technologies are highly important to ensure durability, acceptability by the population and governmental funding.

The earth used in construction is extracted from local soil or can be a waste from excavation works. Both can be used to produce earthen mortars, and they are naturally composed of different fractions and types of clay, silt and sand, after crushing and sieving for the removal of coarser aggregates. The clay is responsible for the agglutinating property in the mixture, acting as a binder. The clay minerals available in nature have different mineral origins and provide different behaviors when used to produce earth mortars, with these being the most common types of clay: kaolinite, illite, montmorillonite and muscovite [2].

Despite providing a general adequate performance in terms of workability, mechanical resistance and adhesion to the substrate, clay is sensitive to the action of water [2]. Therefore, to improve the behavior of earth constructions in relation to the action of rain, many additions have been historically used, such as air lime, vegetable and animal oils and animals’ dung [3].

According to results of project COREMANS [3], to optimize the characteristics of earthen elements, such as hygroscopicity and contribution to hygrothermal comfort, indoor coating mortars (plasters) must be produced exclusively based on clayish earth and sand, with the possibility of adding stabilizing materials in low proportions, but mainly prioritizing those with greater chemical compatibility with the earth components. Furthermore, when building with this type of earth plaster, at the end of its life cycle the plaster can be recycled and used again for new plasters or to produce other earthen products. It can even be used for agricultural cultivation on the construction site itself, for example. Therefore, the choice of stabilizing additions should not alter this positive performance.

Germany shows the importance of earthen products by having had a large set of technical standards for earth building since 2013. Recently, the DIN standards were revised, and some adjustments were made, namely in the DIN 18947 [4] for earth plasters: the possibility of using recycled aggregate; the inclusion of earth adhesive mortar and reinforced mortar. The use of irreversible chemically active binding agents, such as lime, gypsum or cement, remains excluded from the German standard for earth plasters [5].

As a bio-alternative for these irreversible chemically active binder additions for earth building stabilization, in Brazil and in several other regions around the world [6], cow dung is a cow farming residue traditionally used in agriculture but also in vernacular earthen architecture as a stabilizer, for flooring screeds, masonry units, plasters and renders. To this day, its properties and applicability are practiced and recognized in oral popular culture in traditional Brazilian communities [7] and also in other countries.

As environmental issues and organic farming have begun to gain more prominence in recent years, some concerns about the bacteria present in cow dung have also emerged, because cow dung is one of the natural materials that can be used on organic fertilizing. However, cow dung samples have been analyzed and show antimicrobial activity against pathogens [8].

The use of cow dung has been mainly used for agriculture and as a biomass for energy production [9], but other new applications have been tested, such as the use of cow dung fibers for reinforced friction composites [10], the use of adding cow dung ashes on cement mortars and concrete instead of fly ash [11,12] and also the use of adding cow dung on earth building products such as masonry units, mortars and plasters. In fact, despite still being a field lacking research, since the 1990s it has been possible to find some published work on the effects of cow dung additions in earth building. These publications show that, in the correct proportion, the incorporation of cow dung reduces shrinkage [13] and water absorption [14] and increases mechanical strength [15], including adhesion [16], workability and durability [17,18].

To compare the benefits and properties of cow dung with a usual and popular stabilizer addition on earth building, Pachamama et al. [16] investigated and compared the effects of cow dung and an air lime in a Brazilian tropical kaolinitic earth mortar. The cow dung, collected in the pasture, was air dried and turned over to aerate and cool for eighteen days in a big pile, by a traditional farmer. To control shrinkage, four volumetric proportions of sand were added to one volume of earth to produce the reference mortar (0% addition). Mortars with 10% and 20% of cow dung addition, 5% of air lime addition and both 10% of cow dung plus 5% of air lime were formulated. The results showed that the cow dung addition reduced shrinkage and offered more adhesive and mechanical strength than the air lime addition. The mortar with both additions presented better results than the mortar with only air lime, and worse than the mortars with just cow dung.

Millogo et al. [19] and Bamogo et al. [6] clarified that cow dung is rich in nitrogen, phosphorus, phosphoric acid and potassium. When added to kaolinitic earth mortars, these components reacted with kaolinite (Al_2_Si_2_O_5_(OH)_4_) and fine quartz (SiO_2_) present in the clayey earth they tested, resulting in insoluble silicate amines (Si(OH)_4_4NH_3_). This compound united the components of the mixture, providing water resistance and hardness. According to these authors, the fibers present in cow dung also limited the mortars’ shrinkage after drying and reduced the formation of cracks, contributing to increased performance.

Kulshreshtha et al. [18] showed that the addition of cow dung on an earth plastering mortar provided some hydrophobicity; that is, it repelled water on the plaster surface. According to the researchers, this effect was attributed to the presence of microorganisms, bacteria and other products originating from the animal’s digestion process, which reacted with the micro mineral particles present in the earth. The results dialogue with Millogo et al.’s [19] and Bamogo et al.’s [6] conclusions but using different and complementary explanations.

Although these studies present the most complete explanations yet for the effects caused by the addition of cow dung, there are still many doubts regarding the reaction mechanisms between earth and this type of addition. Therefore, it is important to continue investigating the effects of cow dung as a bio-stabilizer on earthen building products, with different chemical and mineralogical compositions, to discover how the benefits of this addition can be optimized.

Contrary to other more standardized materials, soils and the earth extracted from them to be used as a building material are different from one location to another. Between continents, the differences can be more expressive still. Tropical soils and Mediterranean soils are known to be different; the same may also happen with cow dung, as cow species, their climate and food are different. For this reason, it is important to test earth mortars produced with different types of earth and cow dung around the world to check their behavior within a large variety of samples, so that conclusions can be validated.

Therefore, in this study three reference earthen plastering mortars were formulated with the minimal addition of sand to control cracking: one with a Brazilian earth and two with Portuguese earth. Properties were compared between the mortars with earth from the same climatic Mediterranean region and with the tropical earth mortar. The same cow dung and the same percentages were added to the Brazilian and the two Portuguese reference earthen mortar formulations to produce bio-stabilized mortars, and the effects were assessed.

## 2. Materials and Methods

### 2.1. Materials

#### 2.1.1. Earth

**Figure 1:**
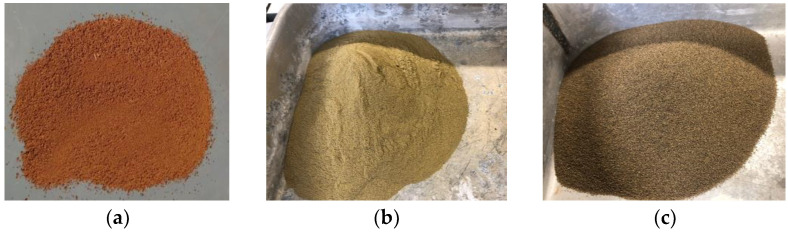
ITA earth (**a**), CAP earth (**b**) and MON earth (**c**) after being sieved, ready to use in mortar production.

The earth used to produce the plastering mortars included one type from Brazil (a tropical earth) and two from Portugal (mediterranean temperate climate earth). The Brazilian earth (ITA) (Figure 1a) was collected in the rural area of Itabirito, on the metropolitan region of Belo Horizonte, in the state of Minas Gerais. The CAP earth (Figure 1b) was collected on the metropolitan region of Lisbon, in Caparica, and the MON earth (Figure 1c) was collected in the rural area of Monsaraz, Alentejo region, both in Portugal.

The three types of earth were manually roughly disaggregated and sieved, passing a mesh with an opening of 2.79 mm. After air drying, the sieved earth was taken to the laboratory to produce the mortars and specimens.

The ITA earth was chosen because it is a common (representative) clay from Minas Gerais, Brazil: it has a large content of clay (70%), mainly kaolinite. The Caparica earth was chosen because it contains a different type of clay, illite, which is very common in Portugal and is siltier. The Monsaraz earth was chosen because it is used with no sand additions to produce traditional handmade solid ceramic bricks with good performance by Telheiro de S. Pedro, Corval, Alentejo, Portugal.

The mineralogical characterization of the earth was performed by X-ray diffraction analysis (XRD), using a Phillips PW3710 X-ray diffractometer with Fe-filtered Co Kα radiation with 35 kV and 45 mA and speed of 0.05 °/s ranging from 3 to 74° 2θ. The minerals were identified by matching the crystalline phases with the International Center for Diffraction Data Powder Diffraction Files (ICDD PDF), and semiquantitative analysis was performed by Rietveld refinement. The mineralogical characterization of the three types of earth used to produce the mortars is presented in Table 1.

The Portuguese CAP earth presents predominance of quartz, followed by feldspars, muscovite and illite. The MON earth presents predominance of feldspars, followed by quartz, hornblende and illite. The Brazilian ITA earth presents predominance of kaolinite, followed by quartz and goethite.

The physical characterization of the earth was determined by particle size distribution, Atterberg limits and loose bulk density. The particle size was determined, using EN 1015-1 [20] and the NBR 7181 [21], by sedimentation for the finer fraction. The Atterberg limits were determined by the NP 143 [22]. The loose bulk density of the materials was determined based on EN 1097–3 [23]. The results are presented in Table 2.

#### 2.1.2. Sand

A siliceous sand (S), basically composed of quartz (SiO_2_), was used. It is a washed medium granulometry sand, with grain size diameter between 0.2 mm and 0.6 mm, commonly used to produce plastering mortars in the area of Lisbon, Portugal. The sand loose bulk density is 1.40 g/cm^3^.

#### 2.1.3. Cow Dung

**Figure 2:**
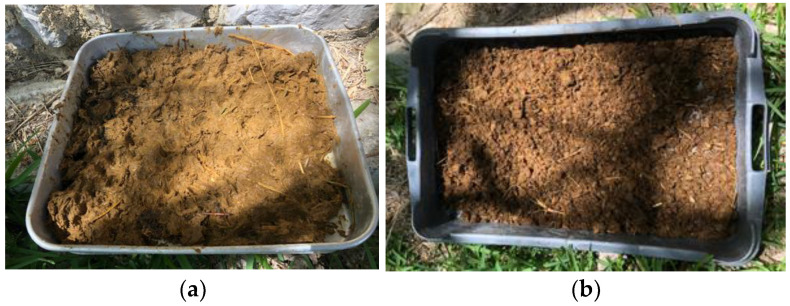
Cow dung freshly collected (**a**) and after drying in an uncovered plastic container for seven days (**b**).

The cow dung (CD) used was collected in the rural area of Moita, a small city in the Lisbon metropolitan area, Portugal. The CD was produced by animals fed on pasture and therefore has many vegetable fibers. It was collected fresh in the pasture (on the same day it was excreted) and left to dry in an uncovered plastic container (Figure 2a) in the shade, with the intention to prevent the chemical liquid component from being lost to the ground. In this drying process, heat, water, methane, carbon dioxide and ammonia are released [6]. Daily, the excrement was turned over to aerate and cool the material. After seven days, the CD had a very light organic odor and some clods, easily manually disaggregated, and it was placed in the laboratory.

While drying, the CD loses water and weight and decreases in density; so it is important to know the moisture content and density when it is used for earth mortar production. The CD moisture content was measured from the day it was collected on the pasture until 60 days of preparation; 10 g was collected for every measurement and placed in an oven at 100 °C for 24 h. When fresh, the CD contained 80–90% wt. of water. After air drying under shade for 7 days, it contained 61% wt. of water (Figure 2b). After air drying in the laboratory for a further 23 days period, it contained only 10–15%. After 60 days, it still contained 10%.

**Figure 3:**
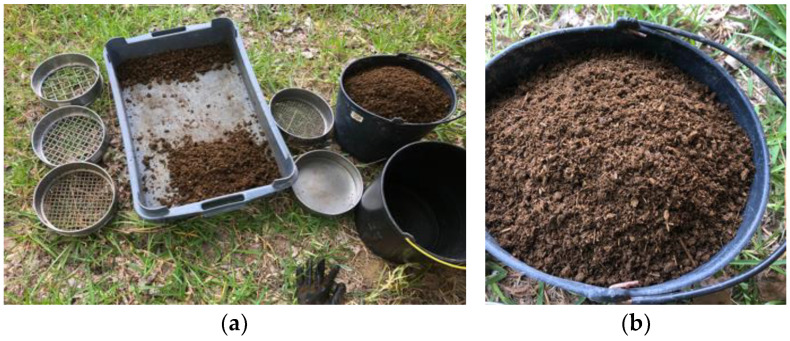
Cow dung being sieved after 7 days of drying (**a**) and after sieving (**b**).

After 7 days drying under shade, the CD was passed through a 2.79 mm sieve (Figure 3a) and became very homogeneous (Figure 3b). A large amount of vegetable fibers, whose length did not exceed 2 cm, could be seen. The density was 0.40 g/cm^2^ and the moisture content was, as mentioned, 61%. Due to this high moisture content, after passing through the sieve some CD particles agglomerated, but were still easy to manually desegregate. Even when the mortars were produced, they did not form clumps with longer lengths and neither were these observed after drying of the plasters applied on the brick and adobe. To produce the mortars, the CD was added to the earth and sand on the same day it was sieved.

According to Millogo et al. [19] and Gupta et al. [9], after being dried the main components of CD are vegetable fibers (essentially composed of cellulose, hemicelluloses and lignin), microorganisms and organic amine compounds (from ammonia that evaporated). Also, there are fragments of intestinal tissues, potassium, phosphoric acid, traces of sulfur, calcium, iron, magnesium and also quartz and clay ingested by the animal accidentally along with grass.

### 2.2. Mortars and Samples

**Figure 4:**
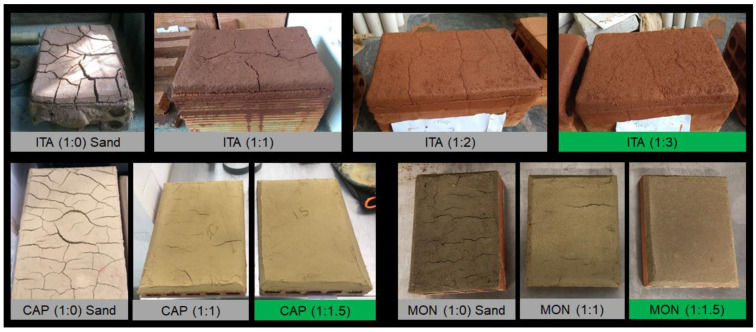
Preliminary shrinkage test to optimize sand addition in ITA (**top**)-, CAP (**down left**)- and MON (**down right**)-earth-based plasters on brick. In green, the chosen optimized earth/sand volumetric proportions.

To define the mortar formulations and the necessary sand addition that allow achieving plastering mortars with limited shrinkage, a preliminary shrinkage/cracking test was performed. Mortars with each type of earth and additions of 0, 1, 1.5, 2 and 3 volumes of sand were produced and used to plaster the surface of ceramic hollow bricks. Comparing the five mortar proportions prepared and applied for each type of earth, the chosen volumetric addition of sand was considered the minimum necessary to minimize the cracks occurring at the surface of the plastered ceramic bricks (Figure 4) after 24 h of air drying on the laboratory environment.

The preliminary shrinkage/cracking test shows that, to limit cracks appearing on the plastering surface, it was necessary to add 3 volumes of sand to 1 volume of ITA and 1.5 volumes of sand to 1 volume of CAP and MON earth mortars. So, these were the ratios of earth/sand used to produce all the mortars—the reference ones (0% of CD) and the bio-stabilized ones with CD additions.

To assess the effect of the CD, earth mortar (earth + sand + water) specimens were produced with each one of the three types of earth with no CD addition, for reference. Two more mortars were formulated with each one of the three reference earth mortars with 10% and 20% of CD additions (in the volume of the dry components: earth plus sand). All mortar compositions are defined in Table 3.

The mortars were produced in a mechanical laboratory mortar mixer based on the instructions of the DIN 18947 [4]. The water was added to reach a flow table consistence of 170 ± 5 mm, ensuring a good workability, and the water/dry components ratio (by mass) is presented in Table 3. The fresh mortars were characterized by the flow table consistence, based on the test procedure described in EN 1015-3 [24] and the fresh density, according to EN 1015-6 [25]. As required by the DIN standard, all fresh mortars had a density greater than 1.2 kg/dm^3^ (Table 3).

**Figure 5:**
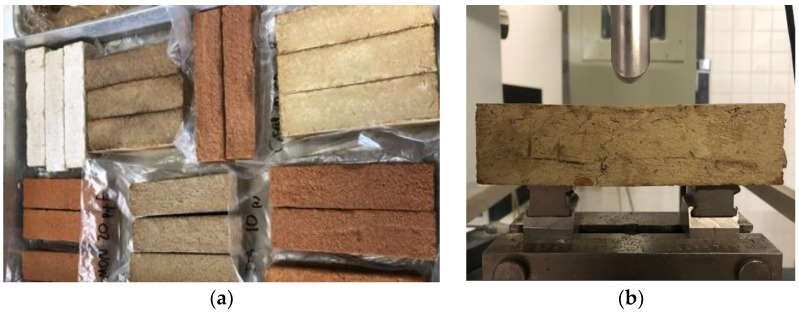
Standard prismatic specimens of the three earthen plasters (**a**); specimen being tested for flexural strength (**b**).

To test the hardened mortars, two kinds of specimens for each one of the 9 formulated mortars were produced. Four standard 40 mm × 40 mm × 160 mm prismatic specimens, as determined in the DIN 18947 [4], were produced per formulation (Figure 5a) in metallic molds. After drying, one of these was cut into three 4 cm × 4 cm × 4 cm pieces and used in the immersion test. The other three were first used for non-destructive tests: linear shrinkage and dry density. Then, they were used for the flexural test (Figure 5b), and the halves for the compressive test.

The specimens were produced and stored in the laboratory at 63 ± 5% relative humidity (RH) and 26 ± 3 °C. These remained in the laboratory environment from their production until the tests were carried out at the age of 30 days. All specimens were tested in equilibrium with the environment (constant mass, as determined by the DIN 18947 [4]).

**Figure 6:**
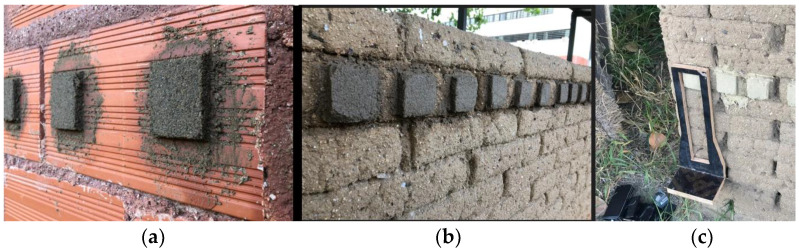
Plaster specimens for adhesive shear strength test: fresh applications on ceramic brick (**a**) and on adobe (**b**) masonry walls, and specimen being tested (**c**).

The second kind of specimen is a plaster one applied on experimental walls (Figure 6) for an adhesive shear stress test, based on Hamard et al. [26]. These have a surface 5 cm × 4 cm and 2 cm thick. Before applying the mortars on the wall, the surfaces were wetted by spraying water. Three specimens were produced for each evaluated mortar on both adobe and hollow ceramic brick masonry walls (Figure 6a,b).

### 2.3. Mortar Test Methods

The tests were performed using the methods and European standards (namely several parts of the EN 1015) recommended by the DIN 18947 [4], specific for earthen plasters. For linear drying shrinkage, three prismatic specimens were used and, according to the DIN 18947 [4], the shrinkage occurring during drying was assessed in percentage by the difference to the molds.

For dry bulk density, the same three prismatic specimens were used and, according EN 1015-10 [27], the density was calculated by the ratio of the mass to the volume, assessed using a digital balance with 0.01 g precision and a digital caliper with precision 0.1 mm.

For flexural and compressive strength, the same three prismatic specimens were used, based on the standard EN 1015-11 [28]. The flexural test was performed first, breaking three specimens per formulation. Then, with the six halves obtained, the axial compression test was performed. For the flexural test, a 2 kN load cell was coupled and configured at a constant speed of 0.2 mm/min on a vertical load application. For the compression test, the 2 kN load cell was maintained and the speed was set to 0.7 mm/min.

For the adhesive shear strength test for each evaluated mortar, three specimens of plastering mortars applied on adobe and three on ceramic brick masonry walls were tested, using the method defined by Hamard et al. [26] and a handmade wooden device, supporting a progressive 250 g increasing mass. The adhesive shear strength (in N/mm^2^) was defined by the maximum mass supported (in g) multiplied by the gravitational acceleration (g = 9.81 m/s^2^) and divided by the adhesive area of the specimen (in mm^2^).

For the immersion in water test, one prismatic specimen per mortar, cut into three 4 cm × 4 cm × 4 cm cubes, was immersed in a 250 mL glass beaker filled with 150 mL of water at the laboratory environmental temperature of 26 °C. Pictures were taken after 1, 2, 5, 10, 15 and 30 min of immersion. Complementary visual observations were also performed after 24 h and 7 and 14 days. An analysis was made about the visual effects observed in the pictures.

## 3. Results

### 3.1. Linear Drying Shrinkage

**Figure 7:**
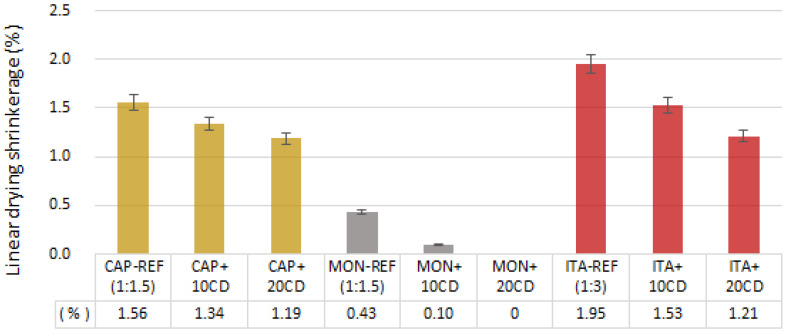
Linear drying shrinkage of the earthen mortars with cow dung (CD) additions and reference mortars: average and standard deviation.

Figure 7 presents the average linear drying shrinkage of the mortars. Although the proportion earth/sand was chosen after a preliminary test (Section 2.2) to reduce the added sand to a minimum, allowing to control the cracking in each reference mortar, the results are significantly different comparing the earthen-based mortars produced with the three different types of earth.

All the mortars comply with the DIN 18947 [4] requirements, with a shrinkage index below 2%, the maximum limit of the standard. Figure 7 shows that the Portuguese earthen mortars, with the same added sand (1.5 of the earth volume) and a similar clay fraction in their composition (around 6%), present a lower linear drying shrinkage than the Brazilian earthen mortars, but which is much lower for the MON mortars. This may be due to several reasons, such as the mineralogical composition of the two types of Mediterranean earth, MON having less expansive compounds, and the higher LL of the CAP earth due to lower silt content. That also justifies why this earth is used to produce handmade traditional bricks without any addition (Section 2.1.1).

The Brazilian earthen mortars’ shrinkage, with double the added sand (3 volumes), is explained by the higher content of clay in ITA earth and consequently on ITA mortar composition (around 18%).

**Figure 8:**
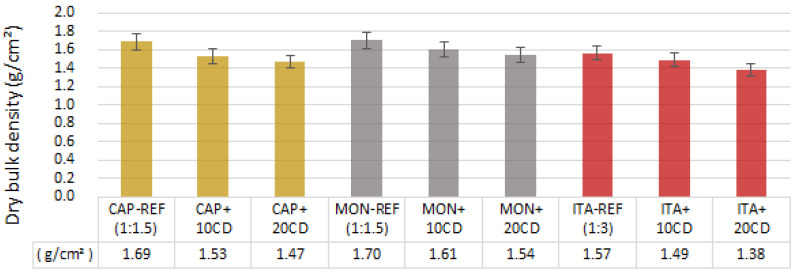
Dry bulk density of the earthen mortars with cow dung (CD) additions and the reference mortars: average and standard deviation.

However, in all the mortars with different types of earth, the addition of CD reduced the linear shrinkage proportionally to the addition (the 20% addition reduced it by more than 10% in all the three earthen mortars). This is most probably explained by the high fiber content of the CD added to the mortars. The other reason is the explanation presented by Millogo et al. [19], Bamogo et al. [6] and Kulshreshtha et al. [18]: the components of CD, whether silicate amines or fatty acids, bind together loose earth particles, which makes shrinkage difficult. However, this binding does not promote a denser structure, as shown below in Figure 8.

The previously referred to researchers also registered a reduction in shrinkage with CD additions, validating the present study results. Bamogo et al. [6] added 0 to 6 wt.% of CD in earth plasters and Millogo et al. [19] added 0 to 3 wt.% of CD on adobe. Both used dried CD powder and observed a reduction in the shrinkage, even with the fibers grinded. The authors attributed this fact to the CD compounds which agglomerated the small, loose particles on the earth.

Looking at the results of linear drying shrinkage (Figure 7), it is evident that the additions of CD significantly reduce the shrinkage of the ITA mortars. Therefore, the CD-added ITA mortars could most probably be further optimized with a reduced addition of sand (1:2 or even 1:1.5 as the CAP and MON mortars), contrary to the ITA reference mortar. The same is true for CD-added CAP and MON mortars: it seems the added sand content could also be reduced in comparison to the reference mortar of both types of earth. The addition of CD would most probably be enough to reduce cracking and shrinkage of mortars. Simultaneously, that would decrease the proportion of sand that had to be specifically extracted to be added, while the earth used could be a construction residue.

### 3.2. Apparent Dry Bulk Density

Figure 8 presents the density of the mortars. The differences between the mortars produced with the different types of earth and the same CD additions are not very significant. However, the MON earth mortars have the highest density, followed by CAP and ITA earth mortars. The addition of CD reduces the density proportionally to the added content in all the three earth mortars. This is correlated with an increase in CD addition because the CD has a density of 0.40 g/cm^2^ and the earth and sand have a density of more than 1 g/cm^2^. Therefore, the higher the amount of CD added, the lower the density of the bio-stabilized mortar will be. So, as expected, the earth mortars with no CD addition (reference mortars) are denser.

The dry bulk density of all mortars has classifiable values regarding the parameters presented by the DIN 18947 [4]: the mortars CAP-REF, CAP+10CD and MON-REF are 1.8 class; the mortars CAP+20CD, MON+10CD, MON+20CD, ITA-REF and ITA+10CD are 1.6 class; and the mortar ITA+20CD is 1.4 class.

### 3.3. Flexural and Compressive Strengths

**Figure 9:**
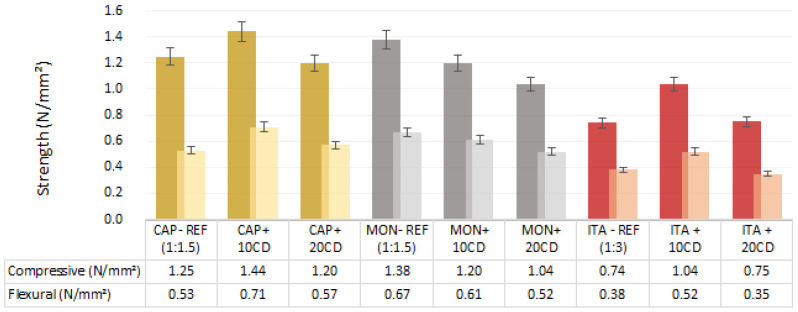
Compressive and flexural strengths of earthen mortars with cow dung (CD) additions and reference mortars: average and standard deviation.

Regarding the flexural and compressive strength test results (Figure 9), the tropical ITA earth mortars present the lowest values in comparison to the temperate climate ones. However, ITA mortars have double the proportion of sand in comparison to the other mortars.

Lima et al. [2] evaluated the mechanical properties of earth mortars produced with the three main types of clay: illite, kaolinite and montmorillonite. In all earth mortars produced with these types of earth, three volumetric proportions of sand were added. The illite earth plasters presented a higher compressive and flexural strength (0.88 N/mm^2^ and 0.25 N/mm^2^, respectively) than the kaolinite earth plaster (0.45 N/mm^2^ and 0.18 N/mm^2^ respectively). Like the present study, the illitic earth mortar showed a higher mechanical strength than the kaolinitic (ITA) one.

Another reason for differences between the results of ITA and CAP/MON may be the geographic climate origin. There are a lot of micro mineral varieties in components that can influence the different flexural and compressive strength performance of mortars produced with tropical and temperate earth. Walter et al. [29] tested the flexural and compressive strength for prismatic specimens of earth mortars with no sand addition produced with 14 different types of tropical earth from French Guiana, presenting a compressive strength from 0.21 N/mm^2^ to 2.53 N/mm^2^ and a flexural strength from <0.3 N/mm^2^ to 0.8 N/mm^2^. This shows the large variation existing just between types of tropical earth. After chemical characterization of the earth, the researchers observed that the mechanical strength of tropical earth mortars had a direct correlation with the earth iron and aluminum oxide contents. In the present study, the ITA tropical earth has an expressive presence of iron oxides, but when it is compared with the two types of temperate climate earth, the mechanical strength is lower than that of the temperate earth. So, there is a combination of factors which contribute to generating the different behaviors between these earth mortars, such as particle size distribution, for example.

Figure 9 provides evidence that, for the CAP and ITA mortars, the addition of 10% of CD increases the strength in comparison to the reference mortars and the mortars with 20% CD. But specifically in the mortars produced with MON, the additions of CD reduce the strength. Despite this, even with the addition of 20% of CD, the strength provided is above the parameters determined by the DIN standard [4], as it stipulates that the compressive strength must be greater than 1 N/mm^2^ and the flexural strength must be greater than 0.3 N/mm^2^.

In earthen mortars produced with the same ITA earth in a 1:4 volumetric ratio earth/sand, Pachamama et al. [16] also observed an increase in flexural and compressive strength with 10% and 20% of another CD addition. The reference mortar, with no CD addition, presented a flexural strength of 0.3 N/mm^2^ and a compressive strength of 0.6 N/mm^2^. The 10% CD addition increased both properties by 50% and the 20% CD addition decreased them by 10%.

In the present research, the values were higher, but this is explained by the higher clay content and probably by the use of fresh CD. In the present study, the 10% CD addition also increased both strengths by 50% in ITA earth mortar. The 20% CD addition did not have a significative influence on the mortar strength in comparison to the reference.

Other authors also register an increase in mechanical strength with CD additions. Millogo et al. [19], when adding 0 to 3 wt.% of CD on adobe, showed an increase in the compressive strength proportional to the increase in the content of CD powder added, with its best performance at an addition of 3%: 2.8 N/mm^2^.

Bamogo et al. [6], when adding 0 to 6 wt.% of CD in earth plasters, evidenced a highest result with a 6% addition, increasing the flexural strength to 0.7 N/mm^2^ and compressive strength to 2.4 N/mm^2^. The researchers attributed this fact to the formation of a new compound (silicate amine) as result of CD and earth interaction. In the present study, the formation of this compound may also agglutinate isolated earth particles together and provide more hardness and strength.

### 3.4. Adhesive Shear Strength

**Figure 10:**
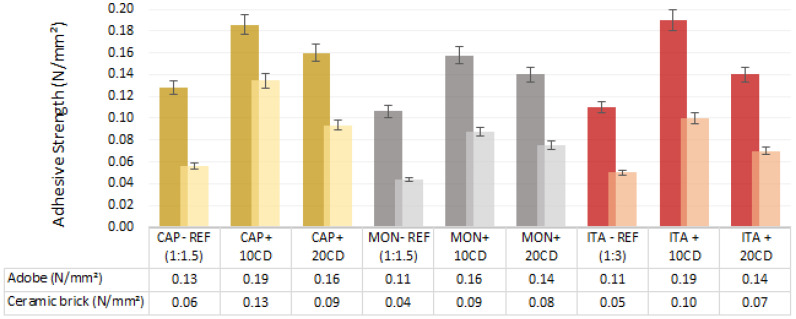
Adhesive shear strength of earthen mortars with cow dung (CD) additions and reference mortars on adobe and ceramic brick masonries: average and standard deviation.

Figure 10 shows that the adhesive shear strength of all the reference earthen mortars is more similar than when comparing the flexural and compressive strength; the difference from the CAP mortar to the ITA mortar is not significant, with the MON mortar being only slightly lower. The results on adobe masonry are always higher in comparison to the ones on ceramic brick masonry.

Furthermore, even with MON, the addition of CD significantly increases the adhesive shear strength (Figure 10). On the ceramic brick masonry, the proportion of 10% CD increased it in 100% on the three types of earth; on the adobe masonry, the same proportion of CD also increased it by more than 50%. The addition of 20% CD also increased the adhesive strength compared to the reference mortars, but this was less expressive.

All mortars present results in accordance with the DIN standard [4] parameters (adhesive strength must be greater than 0.05 N/mm^2^), although the test was performed using a different method than the one defined in the standard.

The increase in the adhesive strength of all earthen plasters with CD to the adobe and ceramic brick masonries is most probably due to the linkage of isolated earth particles provided by the formation of amine silicate, which offers an adhesive property. According to Bamogo et al. [6], the amine silicate molecule behaves as an adhesive because of the free electron pairs on the oxygen atoms and especially on the nitrogen atoms. It seems this adhesive character of the molecule can improve all the mechanical properties of CD-added earthen plastering mortars.

Pachamama et al. [16], when testing the adhesive strength of ITA earth plasters with a different CD addition (from Brazil and more air-dried) and with four volumetric proportions of sand addition instead of three, as in the present study, obtained 0.10 N/mm^2^ for the reference mortar, 0.17 N/mm^2^ for the mortar with 10% CD addition and 0.12 N/mm^2^ for the mortar with 20% CD addition on adobe substrate. In the present study, the results on adobe masonry with a rougher surface were a little higher. This is probably explained by the higher clay content of the plastering mortars, the rougher surface of the adobe and the different test method used.

In the Pachamama et al. [16] study, the standard pull-off test method was used, while in the present one the adhesive shear strength test, according to Hammard et al. [26], was applied because it was considered more adapted and appropriated for earthen plastering, reducing failures during adhesion sample preparation.

Santos et al. [30] evaluated nine earth-based plastering mortars formulated with a 1:3 illitic clayish earth/sand volumetric ratio applied on hollow brick masonry. Using the same method as Hammard et al. [26] and the present study, the researchers found an adhesive shear strength from 0.042 to 0.056 N/mm^2^. Lima et al. [2], evaluating the adhesive strength of earth plasters produced with illitic, kaolinitic and montmorillonitic earth in volumetric ratio 1:3 of earth/sand, applied on hollow ceramic bricks, achieved 0.07, 0.04 and 0.02 N/mm^2^, respectively. The kaolinitic and ilitic earth results of these previous studies are similar to the adhesive strength on ceramic bricks of the reference mortars evaluated in the present research, which were also produced with kaolinitic earth (ITA) and illitic earth (CAP), validating the results.

### 3.5. Immersion in Water

This test is much harder than the usual requirements for earthen plasters, applied indoors and only with contact with water by an open window when it rains, in rooms with water use, such as kitchens and bathrooms, or when cleaning with water. It is also much harder than for renders’ common requirements, as the effect of rain run-off is not so hard as immersion. Therefore, the requirements tested with this method can only be compared when floods occur. Unfortunately, this has been much more frequent recently due to climate change.

**Figure 11:**
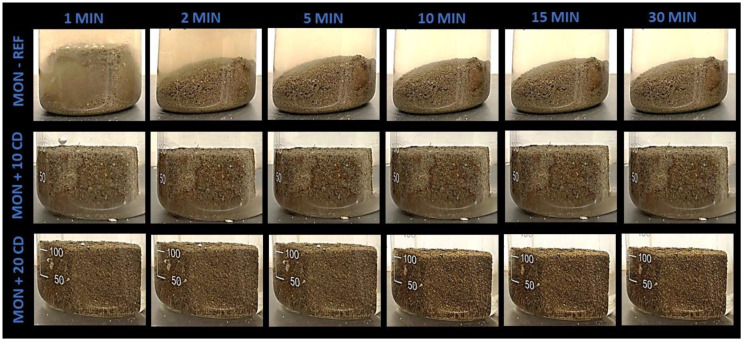
Immersion in water up to 30 min of earthen mortars produced with MON earth, with 10% and 20% cow dung (CD) addition and comparison to the reference mortar.

**Figure 12:**
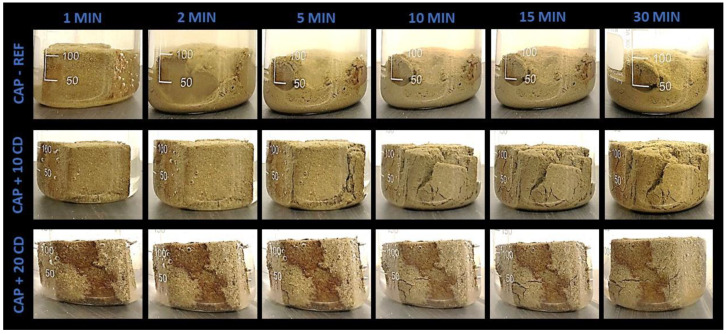
Immersion in water up to 30 min of earthen mortars produced with CAP earth, with 10% and 20% of cow dung (CD) addition and comparison to the reference mortar.

**Figure 13:**
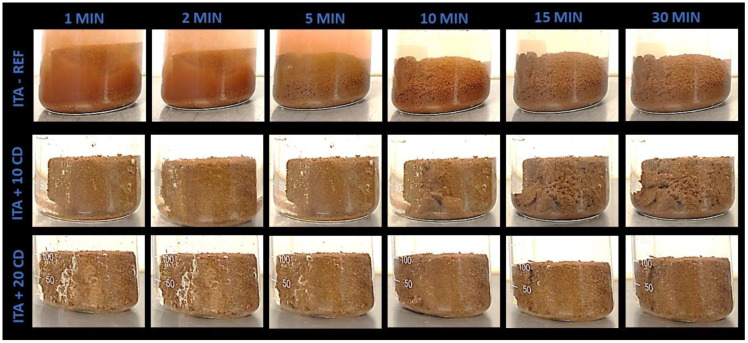
Immersion in water up to 30 min of earthen mortars produced with ITA earth, with 10% and 20% of cow dung (CD) addition and comparison to the reference mortar.

Figure 11 presents the results of the MON-based mortar samples immerged in water after 1, 2, 5, 10, 15 and 30 min. Figure 12 and Figure 13 present the results of the same test performed with the CAP and ITA-based mortars, respectively.

When comparing the resistance to water of the reference mortars, the ITA-REF mortar was the most resistant. In the two minutes picture, the specimen kept the cubic form. The CAP-REF mortar began to disaggregate after 1 min, and after 5 min was almost all turned into powder. The MON-REF mortar was already completely dismantled in 2 min. This different behavior may be explained by the high clay content of ITA earth mortar, which makes it a little more resistant, but probably the mineralogical composition of this tropical earth also has an important role. The MON earth has a similar clay content to the CAP earth, but has more coarse sand and silt. This reason may explain why the MON mortar began to disaggregate quicker than the other two reference mortars.

The 10% CD addition offers greater results in flexural, compressive and adhesive strengths in mortars produced with the three types of earth, but in the water immersion test, the 20% CD proportion presents superior performance compared to the addition of 10%, and substantially superior performance when compared to the reference mortars (Figure 11, Figure 12 and Figure 13). When looking at these figures, the contributions of the additions of CD for the resistance of mortars when immerged in water are significant. The proportion of 20% CD kept the cubic specimen form intact for much longer for all the three CD-added earth mortars.

Curiously, the best behavior under water caused by the CD additions was on the mortars produced with MON earth. Even the proportion of 10% CD already kept the cubic form intact after 30 min of immersion (Figure 11). In addition, after 24 h and 7 and 14 days, the MON+20CD mortar specimens kept the cubic form.

For the mortars produced with CAP and ITA earth, the specimens with 10% CD began to fall apart after 5 min of immersion (Figure 12 and Figure 13). After 30 min, they were already partially disintegrated. The 20% CD mortar specimens kept the cubic form until 30 min of immersion, but cracks began to appear.

Bamogo et al. [6] evaluated the water resistance using two easier test methods: the water absorption test by capillarity and a spray test were used to evaluate the resistance of earth renders to rainfall erosion. The results showed that the capillary water absorption coefficient decreased with CD additions increasing from 0 to 6 wt.%. The spray test showed a diminution of mass loss as CD was added to the mortars. The reference mortar without CD was more eroded. The researchers attribute these results to the presence of insoluble amine silicate, agglutinating isolated earth particles and conferring some hydrophobicity.

Kulshreshtha et al. [18], trying to understand what causes the hydrophobic effect by the addition of 2 wt.% CD to small, compacted earth blocks, carried out the water immersion test and a higher durability of the shape of the specimens produced with CD was clearly observed. The CD used was previously sieved and centrifuged to separate the liquid fraction from the solid fraction (basically vegetal fibers). Both parts were added separately to the compacted block samples and the liquid part offered better effects. For this reason, the hydrophobic effect was attributed to something in the liquid fraction. This liquid suspension, labeled by the researchers as small microbial aggregates, was characterized and “*the results indicated that small microbial aggregates are composed of negatively charged clay-sized particles, rich in short, medium and long chain fatty acids. The short-chain fatty acids in a cow’s intestine are produced through the digestion of dietary fiber by intestinal bacteria (Brody, 1999). One of the dominant fatty acids, octadecanoic acid, has been used with silica nanoparticles to prepare water-resistant super hydrophobic coatings (Heale et al. 2018)*” [18] (p. 6). Therefore, in the present study, fatty acids can most probably explain the improved water resistance of mortars with 10% and particularly 20% CD.

However, the results showed that not only is the CD content important, but as the effect also depends on the earthen mortar formulation, the mineral composition and particle size distribution of the earth are also important. The results also show that the performance of mortars produced with different types of earth may change differently with the same CD addition. Therefore, further studies are needed.

## 4. Conclusions

In the present research, Brazilian tropical earthen plastering mortars (ITA mortars) were compared with two Portuguese temperate climate Mediterranean earthen mortars (CAP and MON mortars) to investigate if significative changes were observed between reference plastering mortars and the effects of two different percentages of cow dung addition. The reference mortars were firstly formulated with the addition of sand content to control their drying shrinkage and cracking, with the Portuguese earth mortars having half the volume of sand added compared to the Brazilian earth mortar.

The tropical earth mortar shows lower mechanical strength, although produced with triple the clay content of the Mediterranean mortars. Probably, this is due to the presence of kaolinite clay, which offers less mechanical strength than illite clay, but also to other differences in the mineral composition and particle size distribution of the three types of earth.

The results show there are differences between the behavior of all three reference mortars. One of the more important differences is that the tropical earth mortar presents a higher durability when immersed in water, probably explained by the higher content of clay of this earth in comparison to the other two tested types of earth; this allows the tropical earth mortar more time in contact with water before beginning to disintegrate. Another is that, as expected, mortars produced with different types of earth, although from not far temperate locations, can perform differently.

The effects of cow dung addition on the hydrophobic properties of the earthen mortars are significant in all the mortars tested, with the 20% proportion (based on the volume of earth plus sand) being more effective than 10%. However, for flexural, compressive and adhesive resistance, the proportion of 10% of cow dung is better than 20%. As the 20% addition also increases the properties in ITA and CAP mortars, it is not a problem to use this higher proportion when the resistance to water is an important requirement.

There may not be an optimized proportion of cow dung, as the most appropriate proportion depends on the application foreseen for the mortar. For example, for coating an external wall with an earthen render, a 20% cow dung addition seems better than 10%. However, the use of that render does not exclude the benefits of a generous overhang on the roof and/or a paint finishing based on limewash, to improve durability. That is also the better choice when considering earthen mortars to repoint uncoated stone masonry walls. On the contrary, to coat indoor surfaces of walls, more exposed to mechanical impacts and less to water, the proportion of 10% of CD seems better than 20%.

The optimization of the earthen mortars by adding cow dung seems to be explained by the fatty acids contained, which provide some hydrophobicity and improve the mechanical performance by agglutinating the earthen particles between them and their adhesion to substrates.

This positive effect of cow dung additions is valid for both the tropical and temperate climate earthen mortars tested. However, in percentage, the benefits of cow dung additions seem more significant when used in the tropical earth. Tropical earth presents different characteristics and, therefore, different performance when compared to temperate climate earth. Furthermore, tropical earth has not been so deeply characterized when used for plasters in comparison to European earth plasters. Therefore, research on earthen mortars produced with tropical earth is needed so it can contribute to generating values appropriate to the reality of many tropical countries, and to composing the parameters of international and tropical countries’ national standards for earthen plasters and renders.

## Figures and Tables

**Table 1:** Semiquantitative analysis (percentage in mass) by XRD of the three types of earth.

Crystalline Compounds	CAP (%)	MON (%)	ITA (%)
Quartz—SiO_2_	51.2	33.6	22.1
Feldspar (microcline)—KAlSi_3_O_8_	9.7	5.2	-
Feldspar (albite)—NaAlSi_3_O_8_	17.5	40.2	-
Mica (muscovite)—KAl_2_(Si_3_Al)O_10_(OH,F)_2_	9.6	-	-
Mica (illite)—(K,H_3_O)(Al,Mg,Fe)_2_(Si,Al)_4_O_10_[(OH)_2_,H_2_O]	8.2	7.1	-
Hornblende—Ca_2_[Mg_4_(Al,Fe^3+^)]Si_7_AlO_22_(OH)_2_	-	13.3	-
Montmorillonite—(Na,Ca)_0,3_(Al,Mg)_2_Si_4_O_10_(OH)_2_.nH_2_O	0.1	0.1	-
Kaolinite—Al_2_Si_2_O_5_(OH)_4_)	0.7	0.1	62.7
Coesite—SiO_2_	-	1.4	-
Goethite—FeO(OH)	-	-	15.2

**Table 2:** Geotechnical and physical characteristics of the three types of earth.

Earth Characteristics	CAP	MON	ITA
Liquid Limit (%)	33	20	54
Plasticity Index (%)	5	3	23
Clay (<0.002 mm)	15	15	70
Silt (0.002 < d < 0.06 mm)	25	40	15
Sand (0.06 < d < 2 mm)	60	45	15
Hygroscopic humidity (%)	2.8	3.8	14
Loose bulk density (g/cm^3^)	1.14	1.34	1.06

**Table 3:** Composition, water/dry components mass ratio necessary to reach 170 ± 5 mm on flow table test and wet density of earth mortars.

Mortars and Volumetric Ratio (Earth/Sand)	Earth (E) Content (% vol.)	Sand (S) Content (% vol.)	Cow Dung Addition (% vol. E + S)	Water/Dry Components (%)	Wet Density (g/cm^3^)
CAP-REF (1:1.5)	40	60	0	20	1.45
CAP+10CD	40	60	10	21.5	1.39
CAP+20CD	40	60	20	23	1.36
MON-REF (1:1.5)	40	60	0	21.5	1.40
MON+10CD	40	60	10	23	1.36
MON+20CD	40	60	20	25	1.31
ITA-REF (1:3)	25	75	0	22	1.85
ITA+10CD	25	75	10	24	1.79
ITA+20CD	25	75	20	26	1.74

## Data Availability

The original contributions presented in the study are included in the article, further inquiries can be directed to the corresponding author.
